# Supplementary material for: Mathematically aggregating experts’ predictions of possible futures
Source: PLoS One. 2021 Sep 2;16(9):e0256919. doi: 10.1371/journal.pone.0256919 (PMC8412308; doi:10.1371/journal.pone.0256919)
Supplement: S1 File — (PDF) [file pone.0256919.s001.pdf]

S1 File

6.1 Preliminary notation

Let N denote the total number of individuals (experts or participants), indexed by $i = 1, \dots, N$ and C denote the total number of events or claims (hereafter, "claim") to be assessed, usually indexed by $c = 1, \dots, C$. Each claim has outcome 1 if the claim is true, and 0 otherwise. For each claim c, each individual i provides assessments that

the claim in question is true or false, by estimating three probabilities: $L_{i,c}$, which is a lower bound ; $U_{i,c}$, an upper bound and $B_{i,c}$ which corresponds to the best estimate for the probability given by individual i for claim c. These estimates satisfy the following inequalities: $0 \leq L_{i,c} \leq B_{i,c} \leq U_{i,c} \leq 1$.

Each claim is assessed by more than one individual and we aggregate their probabilities to obtain a group probability, denoted $\hat{p}_c$. We will further denote $\hat{p}_c\,(Method\ ID)$ as the aggregated probability calculated using the aggregation method with a given ID. For example, the first simple average (arithmetic mean) aggregation for claim c is:

$$\hat{p}_c\,(ArMean) = \frac{1}{N}\sum_{i=1}^{N} B_{i,c} \tag{5}$$

6.2 Weights

Given that many of the aggregation methods proposed involve weighted linear combinations of individual assessments, we can define some standard notation to enhance clarity.

We denote the unnormalized weights by w_method (with subscripts denoting corresponding individuals or claims) and the normalised versions by $\tilde{w}\_method$. All weights need to be normalised (i.e., to sum to one), but as the process is the same for all of them, we will give the formulae for the unnormalized weights. All differentially weighted combinations will take the form:

$$\hat{p}_c\,(Method\ ID) = \sum_{i=1}^{N} \tilde{w}\_method_{i,c} B_{i,c} \tag{6}$$

We note that while most weights will be calculated on a per claim, per individual basis (i.e., judgements from the same individual may be weighted differently on any given claim), in three cases, the weights will be calculated across all claims on a per individual basis only. In these cases, weights for a given individual will not vary across claims and the weights' subscript c from the right hand side of Equation (6) will be dropped.

6.3 Aggregation Methods

Method IDs will simply be abbreviations of the mathematical operations used to calculate the weights.

6.3.1 ArMean: Arithmetic mean of the best estimates

The simplest way to aggregate group estimates is to take the unweighted linear average (i.e., simply takes the average of the best estimates $B_{i,c}$ for each claim). As defined above, the aggregate estimate for claim c is therefore calculated using Equation (5).

6.3.2 Median: Median of the best estimates

Another approach that is often used due to its simplicity is to take the median of the individuals' best estimates.

$$\hat{p}_c\left(Median\right) = Median\left\{B_{i,c}\right\}_{i=1,\ldots,N} \tag{7}$$

6.3.3 LOArMean: Arithmetic mean of the log odds transformed best estimates

Log odds are often used to model probabilities in generalised linear models and state estimation algorithms, typically due to the advantages of mapping probabilities onto a scale where very small values are still differentiable.

$$LogOdds_c = \frac{1}{N}\sum_{i=1}^{N} log\left(\frac{B_{i,c}}{1-B_{i,c}}\right)$$

The average log odds estimate is then back transformed to give a final group estimate:

$$\hat{p}_c\left(LOArMean\right) = \frac{e^{LogOdds_c}}{1+e^{LogOdds_c}} \tag{8}$$

6.3.4 BetaArMean: A beta-transformed arithmetic mean

This method takes the average of best estimates and transforms it using the cumulative distribution function of a beta distribution. This transformation makes the average more

extreme, i.e. increases values larger than 0.5 and decreases values less than 0.5. The Beta distribution is parameterised by two parameters α and β, and in this analysis, we chose $\alpha = \beta$ and larger than one.

$$\hat{p}_c\left(BetaArMean\right) = H_\alpha\left(\frac{1}{N}\sum_{i=1}^{N} B_{i,c}\right) \tag{9}$$

where H_α is the cumulative distribution function of the Beta distribution with two equal parameters.

6.3.5 DistribArMean: Arithmetic mean of the non-parametric distributions

This method assumes that the elicited probabilities and bounds can be considered to represent participants' subjective distributions associated with relative frequencies (rather than unique events). That is to say that we considered that the lower bound of the individual per claim corresponds to the 5% percentile of their subjective distribution on the probability of replication, denoted $q_{5,i}$, the best estimate corresponds to the median $q_{50,i}$, and the upper bound corresponds to the 95% percentile, $q_{95,i}$. With these three percentiles, we can build the minimally informative non-parametric distribution that spreads the mass uniformly between the three percentiles, such that the constructed distribution agrees with participant's assessments and makes no extra assumptions. This approach is inspired by methods for eliciting, constructing and aggregating quantities, rather than probabilities [1].

$$F_i(x) = \begin{cases} 0, \text{ for } x < 0 \\ \frac{0.05}{q_{5,i}} \cdot x, \text{ for } 0 \leq x < q_{5,i} \\ \frac{0.45}{q_{50,i} - q_{5,i}} \cdot (x - q_{5,i}) + 0.05, \text{ for } q_{5,i} \leq x < q_{50,i} \\ \frac{0.45}{q_{95,i} - q_{50,i}} \cdot (x - q_{50,i}) + 0.5, \text{ for } q_{50,i} \leq x < q_{95,i} \\ \frac{0.05}{1 - q_{95,i}} \cdot (x - q_{95,i}) + 0.95, \text{ for } q_{95,i} \leq x < 1 \\ 1, \text{ for } x \geq 1. \end{cases}$$

We then average all such constructed distributions of participants for each claim

$$AvDistribution = \frac{1}{N}\sum_{i=1}^{N} F_i(x);$$

and the aggregation is the median of the average distribution

$$\hat{p}_c\,(DistribArMean) = AvDistribution^{-1}(0.5) \tag{10}$$

6.3.6 IntWAgg: Weighted by interval width

The width of the interval provided by individuals may be an indicator of certainty, and arguably of accuracy of the best estimate contained between the bounds of the interval.We weight according to the interval width across individuals for that claim, defined as follows:

$$w\_Interval_{i,c} = \frac{1}{U_{i,c} - L_{i,c}}$$

$$\hat{p}_c\,(IntWAgg) = \sum_{i=1}^{N} \tilde{w}\_Interval_{i,c} B_{i,c} \tag{11}$$

6.3.7 IndIntWAgg: Weighted by the rescaled interval width (interval width relative to largest interval width provided by that individual)

Because of the variability in the widths of intervals participants give for different claims, we may need to re-scale interval widths across all claims per individual. This results in a re-scaled interval width weight, for individual i for claim c, relative to the widest interval provided by that individual across all claims C:

$$w\_nIndivInterval_{i,c} = \frac{1}{\frac{U_{i,c}-L_{i,c}}{\max\left(\left\{(U_{i,d}-L_{i,d}):d=1,\ldots,C\right\}\right)}}$$

where $U_{i,d} - L_{i,d}$ are individual i's judgements for claim d. Then

$$\hat{p}_c\,(IndIntWAgg) = \sum_{i=1}^{N} \tilde{w}\_nIndivInterval_{i,c} B_{i,c} \tag{12}$$

6.3.8 VarIndIntWAgg: Weighted by variation in individuals' interval widths

A related issue is that participants differ in how much they vary in their interval widths. A higher variance may indicate a higher responsiveness to the existing supporting evidence to different claims. Such responsiveness might be predictive of more accurate assessors. We define:

$$w\_varIndivInterval_i = var\left\{(U_{i,c} - L_{i,c}) : c = 1, \ldots, C\right\}$$

where the variance (var) is calculated across all claims for individual i. Then

$$\hat{p}_c\left(VarIndIntWAgg\right) = \sum_{i=1}^{N} \tilde{w}\_varIndivInterval_i B_{i,c} \tag{13}$$

6.3.9 AsymWAgg: Weighted by asymmetry of intervals

We use the asymmetry of an interval relative to the corresponding best estimate to define the following weights:

$$w\_asym_{i,c} = \begin{cases} 1 - 2 \cdot \frac{U_{i,c}-B_{i,c}}{U_{i,c}-L_{i,c}}, \text{for} B_{i,c} \geq \frac{U_{i,c}-L_{i,c}}{2} + L_{i,c} \\ 1 - 2 \cdot \frac{B_{i,c}-L_{i,c}}{U_{i,c}-L_{i,c}}, \text{otherwise} \end{cases}$$

Then

$$\hat{p}_c\left(AsymAg\right) = \sum_{i=1}^{N} \tilde{w}\_asym_{i,c} B_{i,c} \tag{14}$$

6.3.10 IndIntAsymWAgg: Weighted by individuals' interval widths and asymmetry

Assuming that we want to reward both asymmetric and narrow intervals, we would need to formulate a weight that combines the weights calculated in the *AsymWAgg* and *IndIntWAgg* methods. One simple way of achieving this is to multiply the previously defined and normalised weights.

$$w\_nIndivInterval\_asym_{i,c} = \tilde{w}\_nIndivInterval_{i,c} \cdot \tilde{w}\_asym_{i,c}$$

$$\hat{p}_c\left(IndIntAsymWAg\right) = \sum_{i=1}^{N} \tilde{w}\_nIndivInterval\_asym_{i,c} B_{i,c} \tag{15}$$

6.3.11 KitchSinkWAgg: Weighted by everything but the kitchen sink

KitchSinkWAgg is an ad-hoc method developed and refined using a single dataset (later used in the analysis as well). This method is informed by the intuition that we want to reward narrow and asymmetric intervals, as well as variability between individuals' interval widths (across their estimates).

$$w\_kitchSink_{i,c} = \tilde{w}\_nIndivInterval_{i,c} \cdot \tilde{w}\_asym_{i,c} \cdot \tilde{w}\_varIndivInterval_i$$

$$\hat{p}_c\left(KitchSinkWAg\right) = \sum_{i=1}^{N} \tilde{w}\_kitchSink_{i,c} B_{i,c} \tag{16}$$

6.3.12 DistLimitWAgg: Weighted by the distance of the best estimate from the closest certainty limit

We give greater weight to best estimates that are closer to certainty limits, as follows

$$w\_distLimit_{i,c} = \max\{B_{i,c}, 1 - B_{i,c}\}$$

$$\hat{p}_c\left(DistLimitWAgg\right) = \sum_{i=1}^{N} \tilde{w}\_distLimit_{i,c} B_{i,c} \tag{17}$$

6.3.13 ShiftWAgg: Weighted by judgments that shifted the most after discussion

When judgements are elicited using the IDEA protocol (or any other protocol which allows experts to revisit their original estimates), the second round of estimates may differ from the original first set of estimates an expert provides. Greater changes between

rounds will be given greater weight, with more emphasis on changes in the best estimate such that

$$w\_shift_{i,c} = |B1_{i,c} - B_{i,c}| + \frac{|L1_{i,c} - L_{i,c}| + |U1_{i,c} - U_{i,c}|}{2}$$

$$\hat{p}_c \left(ShiftWAgg\right) = \sum_{i=1}^{N} \tilde{w}\_shift_{i,c} B_{i,c} \tag{18}$$

where $L1_{i,c}, B1_{i,c}, U1_{i,c}$ are the first round lower, best and upper estimates (prior to discussion) and $L_{i,c}, B_{i,c}, U_{i,c}$ are the individual's revised second round estimates (after discussion).

6.3.14 GranWAgg: Weighted by the granularity of best estimates

More skilled forecasters might be expected to have a finer grained appreciation of uncertainty and thus make more granular forecasts.

In our weighting scheme, individuals' received a score of one for each claim that their best estimate was specified at a more granular level than 0.05 (i.e., not a multiple of 0.05), and a zero otherwise. The mean of scores per claim forms a weight per individual, such that

$$w\_gran_i = \frac{1}{C} \sum_{d=1}^{C} \left\lceil \frac{B_{i,d}}{0.05} - \left\lfloor \frac{B_{i,d}}{0.05} \right\rfloor \right\rceil,$$

where $\lfloor\ \rfloor$ and $\lceil\ \rceil$ are the mathematical floor and ceiling functions respectively.

$$\hat{p}_c \left(GranWAgg\right) = \sum_{i=1}^{N} \tilde{w}\_gran_i B_{i,c} \tag{19}$$

6.3.15 EngWAgg: Weighted by the level of engagement as measured by the individuals' verbosity

When assessing claims, individuals have the chance to comment and engage in discussion with other participants. We consider giving greater weight to best estimates that are accompanied by a greater number of comments/justifications. We will consider $w\_eng_{i,c}$

to be the total number of words used by individual i in comments about their estimates for claim c.

$$\hat{p}_c\,(EngWAgg) = \sum_{i=1}^{N} \tilde{w}\_eng_{i,c} B_{i,c} \tag{20}$$

6.3.16 ReasonWAgg: Weighted by the breadth of reasoning provided to support the individuals' estimate

When individuals provide multiple unique reasons in support of their judgment, this may indicate a breadth of thinking, understanding and knowledge about the claim and its context, and may also reflect engagement and conscientiousness. Giving greater weight to best estimates that are accompanied by a greater number of supporting reasons may be beneficial. We will consider $w\_reason_{i,c}$ to be the number of unique reasons provided by that individual i in support of their estimate for claim c.

$$\hat{p}_c\,(ReasonWAgg) = \sum_{i=1}^{N} \tilde{w}\_reason_{i,c} B_{i,c} \tag{21}$$

Qualitative statements made by individuals as they evaluate claims/studies were coded by the repliCATS Reasoning team, according to a detailed coding manual developed to ensure analysts were each coding for common units of meaning in the same sets of textual data. This manual emerged through an iterative process, that included calculating the inter-coder-reliability (ICR), in the form of Krippendorf's alpha [2]. Roughly, ICR measures the extent to which different judges assign similar ratings to the evaluated characteristics, here in the form of reasoning categories. For context, an ICR (here Krippendorf's alpha) of 1 indicates perfect reliability, while 0 indicates the absence of reliability. Values less than 0 indicate systematic disagreements. From this manual, a subset of 25 codes were selected as reasoning categories, each of which were included in ReasonWAgg if the ICR was calculated at a minimum of 0.66 across two or more analysts, or an ICR between two analysts of at least 0.75 and a minimum overall ICR of 0.50. A quarter of the dataset was manually coded into these categories by multiple analysts (using the NVivo Qualitative Data Analysis Software, Version 12, 2018), and

these datasets provided the training data for the remaining text to be auto-coded in NVivo. Reasoning scores were calculated for individuals who received one point for each of these 25 reasoning categories that they drew on over the course of their evaluation (i.e., statements from both IDEA rounds). The reasoning categories include: the plausibility of claim, effect size, sample size, presence of a power analysis, transparency of reporting, journal reputation.

ReasonWAgg can be modified to incorporate not only the number of reasons, but also their diversity across claims. This modified aggregation will be called ReasonWAgg2. The latter component of this score will be calculated per individual from all the claims they assessed, so it will be the same for each of the claims assessed by that individual. We assume each individual answers at least two claims. If a participants has assessed only one claim, for that claim we will default to the original ReasonWAgg.

Table 3 shows a hypothetical example of the reasons used by one participant when assessing four claims.

Table 3: The distribution of the reasons one participant mentioned in the comments they made when assessing four claims. A $(Claim, R)$ cell is 1 if the R was used to justify answers for $Claim$, and empty if R was not mentioned.

$Claims/Reasons$	R_1	R_2	R_3	...	R_{25}	Weighted "No. of Reasons"
$Claim_1$	1			1		$0.75 \cdot 1 + 1 \cdot 0.5 = 1.25$
$Claim_2$		1				0.75
$Claim_3$			1			0.5
$Claim_4$			1	1		1
Av. use of R_r	$\frac{1}{4}$	$\frac{1}{4}$	$\frac{2}{4}$	$\frac{2}{4}$	$\frac{0}{4}$	
1 - Av. use of R_r	0.75	0.75	0.5	0.5	0	

The penultimate row of the matrix showed in Table 3 gives the average use of reasons, and the last row shows weights assigned to these reasons. These weights are then used to calculate a final reasoning score per claim (per participant). This score is showed in the last column of Table 3 and it is calculated as a weighted sum of the elements of the vector of zeros and ones indicating use of reasons per claim.

We will consider $w\_varReason_{i,c}$ to be the weighted "number of unique reasons" provided by participant i in support of their estimate for claim c. Assume there are

25 unique reasons any participant can use to justify their numerical answers. Then, for each participant i we can construct a matrix $\mathbf{CR_i}$ with 25 columns, each corresponding to a unique reason, r, and C rows, where C is the number of claims assessed by that participant. Each element of this matrix $\mathbf{CR_i}(r,c)$ can be either 1 or 0. $\mathbf{CR_i}(r,c) = 1$ if reason R_r was used to justify the estimates assessed for claim c, and $\mathbf{CR_i}(r,c) = 0$ if reason R_r was not mentioned when assessing claim c.

$$w\_varReason_{i,c} = \sum_{r=1}^{25} \mathbf{CR_i}(c,r) \cdot \left(1 - \frac{\sum_{c=1}^{C} \mathbf{CR_i}(c,r)}{C}\right)$$

ReasonWAgg2 will use the weights calculated as above in the prediction given by Equation 21.

6.3.17 QuizWAgg: Weighted by performance on the quiz

As part of the repliCATS project, individuals were asked to take a quiz before commencing the main task of evaluating research claims. Hence, this aggregation method will only apply to a dataset where such an exercise is undertaken prior to the elicitation. The quiz aimed to gauge subject matter expertise, and in the case of the repliCATS project, consisted of questions testing familiarity with previous research and concepts related to assessments of replicability, e.g., understanding of statistical concepts, (false) positive rates and replication rates in domain-relevant literature, and self-reported rates of questionable research practices. If answered, quiz responses would provide similar information to that of seed questions, enabling differential performance-based weighted combinations (giving greater weight to individuals with higher quiz scores). The quiz was encouraged, but not compulsory, so choosing to take the quiz at all may also reflect engagement and conscientiousness.

The quiz contains $n_{quiz} = 22$ questions, 12 of which cover knowledge and understanding of statistical concepts, and 10 are about meta-research. Questions that required less effort to answer (i.e., 10 true/false questions and one two-part question) were assigned half points.

Individuals provide answers for each question, resulting in a $N \times n_{quiz}$ matrix $\mathbf{Q}$, where

each element $\mathbf{Q}(i,h)$ is 1 if individual i answered question h correctly, and 0 otherwise. For each question answered correctly, the individual receives points, with the number of points received for a correct answer for each of the 22 questions specified in the points vector

$$\mathbf{v} = \begin{cases} 0.5, & \text{for questions 1 to } 10, 16, 17 \\ 1, & \text{for questions 11 to } 15, \text{and} \quad 18 \text{ to } 22 \end{cases}$$

This results in a quiz score that ranges from 0 to 16, with higher scores indicating better performance. Then the un-normalised weight based on the quiz score is

$$w\_quiz_i = \mathbf{Q} \cdot \mathbf{v}$$

and the aggregated estimate is

$$\hat{p}_c\left(QuizWAgg\right) = \sum_{i=1}^{N} \tilde{w}\_quiz_i B_{i,c} \tag{22}$$

where w_quiz_i is the weight corresponding to the score of individual i on the quiz, as defined above.

In the case of unanswered questions (missing data), individuals are assigned zero points for that question. Individuals who did not take the quiz at all will receive zero weight (and non-zero weight for those who responded to at least one item in the quiz). If only one person assessing a given claim took the quiz, the $QuizWAgg$ aggregated estimate for that claim will be based solely on their judgment. If, however, nobody took the quiz, this aggregation method is impossible to construct.

6.3.18 CompWAgg: Weighted by the level of self-rated comprehension of the claim the individuals' report

In the repliCATS project, before assessing a claim, individuals were asked to assess how well they understood it. A 7-point scale, where 1 corresponds to "I have no idea what it means" and 7 corresponds to "It is perfectly clear to me" is used for this comprehensibility question. Intuitively, the numerical estimates of the individuals who are confident they

understood the claim may be weighted more. We will consider $w\_comp_{i,c}$ to be the number assigned to the comprehensibility, as provided by individual i in support of their estimate for claim c.

$$\hat{p}_c\left(CompWAgg\right) = \sum_{i=1}^{N} \tilde{w}\_comp_{i,c} B_{i,c} \tag{23}$$

6.3.19 BayTriVar: Bayesian Triple-Variability Method

The last two aggregation methods proposed are Bayesian methods, and hence they use the elicited probabilities differently, namely as data with which prior distributions are updated.

Three kinds of variability around best estimates are considered: generic claim variability, generic participant variability, and claim - participant specific uncertainty (operationalised by bounds). The model takes the log odds transformed individual best estimates as input (data), uses a normal likelihood function and derives a posterior distribution for the probability of replication. That is to say, the log odds transformed best estimate data are assumed to follow a Normal distribution $log\left(\frac{B_{i,c}}{1-B_{i,c}}\right) \sim N(\mu_c, \sigma_{i,c})$, where μ_c denotes the mean estimated probability of replication for claim c, and $\sigma_{i,c}$ denotes the standard deviation of the estimated probability of replication for claim c and individual i. Parameter $\sigma_{i,c}$ is calculated as:

$$\sigma_{i,c} = (U_{i,c} - L_{i,c} + 0.01) \times \sqrt{\sigma_i^2 + \sigma_c^2}$$

with σ_i denoting the standard deviation of estimated probabilities of replication for individual i and σ_c denoting the standard deviation of the estimated probability of replication for claim c. The above formula for the standard deviation is derived using the statistical rules for calculating the variances of a sum of two independent random variables. The distribution of the best estimates is considered to be the convolution of the claim and participant distributions (thought of as independent). The sum of these two variables is then scaled by a constant (the width of an interval for a particular claim) which represents the claim - participant specific uncertainty. The variance then is the scaled addition of

the two variances.

To complete the specification of the Bayesian model, priors need to be given for μ_c, σ_i, and σ_c. These are defined as $\mu_c \sim N(0, 3)$, $\sigma_i \sim U(0, 10)$ and $\sigma_c \sim U(0, 10)$, with $U(0, 10)$ denoting the Uniform distribution on the interval from 0 to 10. The quantity of interest is the median of the posterior distribution of μ_c, the mean estimated probability of replication. In Bayesian statistics the posterior distribution is proportional to the product of the likelihood and the prior and in this instance a Monte Carlo Markov Chain algorithm [3] is used to sample from this posterior distribution. After obtaining the median of the posterior distribution of μ_c, we can back transform to obtain $\hat{p}_c$:

$$\hat{p}_c\left(BayTriVar\right) = \frac{e^{\mu_c}}{1 + e^{\mu_c}} \tag{24}$$

6.3.20 BayPRIORsAgg: Prior derived from predictive models, updated with best estimates

This BayPRIORsAgg method uses Bayesian updating to update a prior probability of replication estimated from a predictive model with an aggregate of the experts' best estimates for any given claim. The main difference between this method and the one presented in Section 6.3.19 is that the parameters of the prior distribution of μ_c are informed by the PRIORS model [4] which is a multilevel logistic regression model that predicts the probability of replication using attributes of the original study.

References

[1] Cooke RM. Experts in uncertainty: Opinion and subjective probability in science. Environmental Ethics and Science Policy Series. Oxford University Press; 1991.

[2] Krippendorff K. Content Analysis: An Introduction to Its Methodology, 4th Edition. Los Angeles: SAGE Publications; 2019.

[3] Ravenzwaaij vD, Cassey P, Brown SD. A simple introduction to Markov Chain Monte–Carlo sampling. Psychon Bull Rev. 2018;25:143–154.

[4] Gould E, Wilkinson DP, Willcox A, Groenewegen R, Vesk P, Fraser H, et al. Using model-based predictions to inform the mathematical aggregation of human-based predictions of replicability. MetaArXiv Preprints. 2021; Available from: https://doi.org/10.31222/osf.io/f675q.
