# Supplementary material for: Mathematically aggregating experts’ predictions of possible futures
Source: PLoS One. 2021 Sep 2;16(9):e0256919. doi: 10.1371/journal.pone.0256919 (PMC8412308; doi:10.1371/journal.pone.0256919)
Supplement: Attachment — Submitted filename: Review.pdf [file pone.0256919.s002.pdf]

Review Report on "Mathematically aggregating experts' predictions of possible futures"

The authors consider the problem of aggregating experts' probability predictions of a future binary outcome. They consider many different aggregators that derive from the weighted average and differ in the way the weights are calculated. They apply the aggregators to three real-world datasets and compare the performances of the aggregators in terms of several well-established criteria. Even though no single aggregator emerges as a clear winner, the beta-transformed arithmetic mean (BetaArMean) performs very well.

Overall, I enjoyed reading the paper, and I believe the literature would benefit from an extensive comparison of different weighting schemes. However, the paper needs work before being published. I have listed below both general and specific comments. I hope they help the authors to improve this paper.

General Comments

1. Many of the weighting schemes are stated without providing any rationale or evidence from previous literature that such a scheme could be reasonable. The authors should put in more effort to explain the intuition behind each scheme and, whenever possible, cite past work that has argued for such a scheme.

2. The framing needs work. Currently the text is 33 pages long and feels like a long enumeration of different weighting schemes. Furthermore, the discussion and results only take up around 5 pages. Perhaps the following could help:

   (a) Introduction: Include a paragraph or two clearly stating what the goal of this study is. One idea is to state that unequal weights based on seed variables have been found to improve averaging in the context of point forecasts. This article now studies whether the result holds under probability forecasts. This is roughly what you already state in the beginning of Section 3. This statement, however, should come much earlier in the paper.

   (b) Discussion: What is the main take-away or discovery in this paper? If you decide to use the study goal proposed above, then you should link the results and discussion to that. For instance, can you say anything about why different weighting schemes make little difference in the context of probability predictions? What makes this inherently different from point

predictions? Can you propose a solution for making the probability forecasting context more like the point forecasting context?

Specific Comments

1. Line 75: The authors could consider the work on *the Wisdom of the Inner Crowd* that has found improved accuracy from averaging multiple predictions from the same individual. See, e.g., Herzog and Hertwig (2014).

2. Line 151: There is a typo: "outcomr"

3. Line 151: The authors could give context by stating that a score of 0 implies perfect accuracy and 0.5 is guaranteed by constantly predicting 0.5 for all future events.

4. Lines 174-175: The authors mention "arbitrary probability threshold." Can you explain what this means? The AUC requires a threshold (e.g., 0.5) above which the event is taken to occur and below which it is taken to not occur. How is this done exactly in your case?

5. Equation (4): Can you define $I(p_k, 0.5)$? Perhaps I missed it.

6. Lines 251-255: The authors claim that more seed variables are needed when probabilities are elicited. Can you provide intuition for this? My intuition says the opposite: by asking the experts' for their full distribution – not just a point estimate – I gain more information about their true ability per prediction. This suggests that I would need fewer – not more – seed variables.

7. Lines 269: The phrase "In formulae where we single out claim c, we will have claims indexed by d, for $d = 1, \ldots, C$" is confusing. First you index claims by c, but then also mention d. Perhaps a simple (one sentence) example can help here to clarify.

8. Lines 275: The authors should consider adding a table with summary statistics of the data. For instance, this could include the number of claims, number of experts, number of predictions per claim, the number of claims that were true (a.k.a., the base rate).

9. Section 2.9: The weighted average of probability prediction is known to lack both calibration and sharpness even if the individual probabilities are calibrated. Ranjan and Gneiting (2010) show this for fixed weights. Satopää (2017) generalize the result to random weights. This sub-optimality then led to the development of the extremizing algorithms (see, e.g., Satopää et al. 2014; Baron et al. 2014). The authors should, in particular, review Baron et al. (2014) because it can help them motivate the median and some of the other aggregators. Now, these sub-optimality results are theoretical and they do not imply that the weighted average can never be useful. There are papers arguing the equally weighted average is "robust" in the sense that it does not rely on unstable parameter estimation (e.g., Jose and Winkler 2008). As a result, it can be useful. Finally, it is interesting that the authors do not cite Ranjan and Gneiting (2010) even though, I believe, this is where the BetaArMean aggregator was originally proposed.

10. Line 310: Sometimes the probit instead of the logit is used. These two link functions are, however, similar when the predictions are close to 0.5. But both become infinitely large or small if the probabilities are 1.0 or 0.0. How did the authors handle such extreme predictions?
11. Line 313: To what papers are the authors referring here? Can you include citations?
12. Line 337: The authors could provide more intuition or citations to back up some of the weighting schemes. For instance, what is the intuition behind IndIntWAgg? It seems that it could be argued on the grounds of individuals having different perceptions of the probability scale. Is this accurate? Second, consider AsymWagg. Why is asymmetry a sign of certainty? Any citations supporting this claim?
13. Lines 368-370: This sentence is confusing. I believe $r = 0.33$ is the correlation coefficient but based on the given wording I cannot be sure.
14. Line 402: Why limit to granularity threshold of 0.05? The authors could consider several levels of granularity: 0.1, 0.05, and 0.01. The more granular the prediction, the more weight it gets.
15. Line 427: Does the number of reasons correlate with accuracy?
16. Lines 423-434: What is ICR? Can the authors explain this part more carefully?
17. Line 470: Does this mean that the accuracy scores (Brier, AUC, etc.) shown for QuizWAgg are based on different set of outcomes than the rest of the aggregators?
18. Line 481: What is the rationale for giving the points in this manner?
19. Line 504: The authors could use a figure to illustrate $F_i(x)$.
20. Line 509: Can you explain why you have chosen this form for $\sigma_{i,c}$? What is the motivation for it?
21. Line 520: Instead of calculating the posterior mean of μ_c and then transforming it to the unit interval, you could propagate the posterior uncertainty to the aggregate $\hat{p}_c$. In other words, the proper Bayesian way of doing this would compute $\hat{p}_c$ for each one of the posterior draws in your MCMC sample and then use the mean of those values as the final aggregate.
22. Line 527: The "[link to repository]" does not seem work.
23. Move Section 4 before Section 2. This way you can use the data as a motivation, and the descriptions of the aggregators would make more sense as well.
24. Have the authors made the repliCATS and ACE-IDEA datasets publicly available? I did not see a mention of this in the paper.

25. Line 622: The GJP also considered teams (both trained and untrained) and superteams. Each team had around 5 individuals. In the publicly available GJP dataset, the untrained teams are indicated by condition 4a; the untrained teams with condition 4b; and the superteams with condition 5. Perhaps it makes more sense to analyze these groups rather than "random groups" of 10 individuals.

26. In the ACE tournament the experts were allowed to make and update their predictions until the events resolved. Of course, predicting an event 3 months from now is much harder than something that will happen tomorrow. How did the authors take into account the different time horizons of the predictions? As a suggestion, they may consider using predictions made 30 days before the event resolutions, as was done in Satopää et al. (2021).

27. Figure 1: Could the authors comment on the statistical significance of these differences? For instance, the repliCATS is a very small dataset. Therefore it is likely that none of these differences are statistically significant.

28. There are some inconsistencies in the discussion. For instance, on line 673 the authors state that "there are very few reasons to proclaim one aggregation method better than another." But then on line 690 the BetaArMean is declared as a "clear winner."

29. Lines 712-720: I liked this discussion on the median. There are several papers arguing that the median performs well. For instance, consider Hora et al. (2013) and Han and Budescu (2019). Perhaps these references can help the authors to strengthen this result.

30. An interesting future study could compare the different types of *augmented elicitation* schemes where the decision-maker asks the experts to provide more than just the prediction of the outcome. Recent references are Prelec et al. (2017); Palley and Soll (2019); Palley and Satopää (2020); Martinie et al. (2020).

References

Baron, J., Mellers, B. A., Tetlock, P. E., Stone, E., and Ungar, L. H. (2014). Two reasons to make aggregated probability forecasts more extreme. *Decision Analysis*, 11(2):133–145.

Han, Y. and Budescu, D. (2019). A universal method for evaluating the quality of aggregators. *Judgment and Decision Making*, 14(4):395.

Herzog, S. M. and Hertwig, R. (2014). Harnessing the wisdom of the inner crowd. *Trends in cognitive sciences*, 18(10):504–506.

Hora, S. C., Fransen, B. R., Hawkins, N., and Susel, I. (2013). Median aggregation of distribution functions. *Decision Analysis*, 10(4):279–291.

Jose, V. R. R. and Winkler, R. L. (2008). Simple robust averages of forecasts: Some empirical results. *International journal of forecasting*, 24(1):163–169.

Martinie, M., Wilkening, T., and Howe, P. D. (2020). Using meta-predictions to identify experts in the crowd when past performance is unknown. *Plos one*, 15(4):e0232058.

Palley, A. and Satopää, V. (2020). Boosting the wisdom of crowds within a single judgment problem: Selective averaging based on peer predictions. *Available at SSRN 3504286*.

Palley, A. B. and Soll, J. B. (2019). Extracting the wisdom of crowds when information is shared. *Management Science*, 65(5):2291–2309.

Prelec, D., Seung, H. S., and McCoy, J. (2017). A solution to the single-question crowd wisdom problem. *Nature*, 541(7638):532–535.

Ranjan, R. and Gneiting, T. (2010). Combining probability forecasts. *Journal of the Royal Statistical Society: Series B (Statistical Methodology)*, 72(1):71–91.

Satopää, V. A. (2017). Combining information from multiple forecasters: Inefficiency of central tendency. *arXiv preprint arXiv:1706.06006*.

Satopää, V. A., Baron, J., Foster, D. P., Mellers, B. A., Tetlock, P. E., and Ungar, L. H. (2014). Combining multiple probability predictions using a simple logit model. *International Journal of Forecasting*, 30(2):344–356.

Satopää, V. A., Salikhov, M., Tetlock, P. E., and Mellers, B. (2021). Bias, information, noise: The bin model of forecasting. *Management Science*.
