# Supplementary material for: Mathematically aggregating experts’ predictions of possible futures
Source: PLoS One. 2021 Sep 2;16(9):e0256919. doi: 10.1371/journal.pone.0256919 (PMC8412308; doi:10.1371/journal.pone.0256919)
Supplement: Attachment — Submitted filename: Review.pdf [file pone.0256919.s004.pdf]

Review Report on “Mathematically aggregating experts’ predictions of possible futures”

The authors consider the problem of aggregating experts’ probability predictions of a future binary outcome. They consider many different aggregators that derive from the weighted average and differ in the way the weights are calculated. They apply the aggregators to three real-world datasets and compare the performances of the aggregators in terms of several well-established criteria. Even though no single aggregator emerges as a clear winner, the beta-transformed arithmetic mean (BetaArMean) performs very well.

The paper has improved noticeably through the revision: The paper is clearer and now reads more like a research paper, instead of a long enumeration of different weighting schemes. The authors have added reasons for the different aggregators, placed them into three groups, and discuss the results more coherently than before. Most of my concerns were addressed. Only a few concerns remain. See below for an enumeration of comments.

Comments

1. I like how the authors have brainstormed for many different weighting schemes and how they then compare the aggregators on several datasets on important applications. However, having read the paper a few times now, I am still left thinking: ”What have I learned? What do I know now that I did not know before?” I would encourage the authors to add in the discussion section one paragraph that states this clearly.

2. Figure 2 and Table 2 are great new additions!

3. The final paragraph on page 4. The authors explain how probability predictions require more seed variables than quantile predictions. I had an earlier comment on this as well, and unfortunately it is still not entirely clear to me. Is it because with probability predictions a forecaster reports only one quantity per outcome, whereas with quantile predictions the forecaster reports multiple quantities (the quantiles at given cutoffs), leading to more stable evaluation of accuracy? I hope the authors can help me understand this better, either in response or in the paper.

4. Page 8, line 215: I thought this was the definition of calibration/reliability – not of resolution.

5. Page 13, lines 334-335: It seems that the authors use each forecaster's final prediction of an outcome. In the GJP dataset, some forecasters updated frequently, some very little. Some questions were open for months, others for weeks. The authors way of choosing the predictions can result in situations where one forecaster's prediction was made months before the event resolution (with high amounts of uncertainty) while another forecaster's prediction was made a day before the event resolution (when almost no uncertainty remains). Clearly the forecasts were made under very different circumstances. I made an earlier comment on this as well and suggested that the authors would use a 30-day horizon. The authors, however, chose not do this because in the replication study time is difficult to define and use. Unfortunately, I am not convinced by this argument. The different studies use different questions/contexts anyways. I assume that in the replication study the forecasters made predictions under roughly similar levels of uncertainty. Shouldn't the authors seek to control for the level of uncertainty in their analysis of the GJP data as well?

6. I like how the authors now group the aggregators. This is very helpful. The authors could also discuss how the aggregators have different data requirements. For instance, extremization needs past performance data, whereas the median does not. Therefore, it is not surprising to see that extremization performs better than the median. Conditional on similar data requirements and hence breadth of applications, which aggregators seem to perform the best? This could be added, e.g., into the discussion.
