# Supplementary material for: Mathematically aggregating experts’ predictions of possible futures
Source: PLoS One. 2021 Sep 2;16(9):e0256919. doi: 10.1371/journal.pone.0256919 (PMC8412308; doi:10.1371/journal.pone.0256919)
Supplement: Attachment — Submitted filename: Answers2Comments.docx [file pone.0256919.s003.docx]

***Note to the editors***

***To address the reviewers’ comments, we have reorganized the existing text, added extra text, added a couple of tables, a figure and Supporting information. As a consequence, the paper changed considerably and became considerably larger.***

***We have tried our best to signal the changes by marking them in red. The sections for which the text was slightly augmented, but mostly reorganized are marked in red in their entirety, since the flow changed. From Section 3 until Section 6 most of the text changed, so everything is marked in red. Most of what constituted Section 3 in the previous version is now Section 6. Slight modifications of the text and additions in what is now Section 6 are marked in red as well. New figures and tables were added, and if they are surrounded by red text it means they are new.***

***We have answered the reviewers’ comments inline (formatted in bold, italic) below.***

**Reviewer #1**

This is a very nice work, I like reading it very much. The way it is written is really clear, and using an astounding English writing that I am unable to judge. I found the paper very informative in terms of a "Review" of the different aggregation methods that can be used from experts' predictions.

***Thank you!***

1) I can perform two main criticism of this work. The first one is that, though it may be influenced from my background, I think the manuscript can benefit if it is more connected to Computer Science concepts. For instance, I found that the provided definition for Entropy is not the standard definition given in computer science or statistical sciences.

***The entropy concept, which was mentioned in the previous version of the paper is the entropy of a discrete random variable, which measures the "uncertainty" inherent in the variable's outcomes. It is also known as Shannon’s entropy and in a way, it is equivalent with Gibbs’ entropy from statistical mechanics. The informativeness measure used in the analysis is based on the entropy. However, we deleted the references to entropy to avoid confusion.***

2) The second criticism is that the evaluation of the 22 methods lack any statistical proof about the obtained results. I am not pointing towards some extreme statistical hypothesis test but at least some paragraph stating what kind of control experiments authors executed to verify that obtained results are not affected by some uncovered bias. For instance, what happens if Authors run the 22 tests on an artificial dataset that is produced by an agent that randomly produces responses? I do not think that per se that invalidates anything about this work, but anyway, it would beneficial to understand if the results that Authors finally obtained are affected by chance or not.

***The analysis and comparisons between the methods are performed on real data, collected from three experiments. Simulated data was not used in this case. We have added text to specify and justify these on page 5 (lines 136 - 139) . We have also described the three data collection experiments in greater detail.***

Some minor issues that I found on the manuscript:

3) 43-45: Are Authors suggesting that when the relative frequency interpretation is not appropriate, the reason why it should be encouraged to elicit bounds while making a probabilistic forecasting decision is because it helps the expert to elucidate a best estimate B i,c ?

***Yes, that is what we are suggesting.***

4) 104: I suggest authors that they may link the idea of analyzing or considered prior performance in a similar domain, connected with the concept of transfer learning in Computer Science/Machine Learning.

***Thank you for the suggestion; we have added text and a reference on page 5, lines (118 -121).***

5) 157: Equation (1): I would like to suggest Authors to use shorter variable names in Equations, or greek symbols. The mathematical notation using very long variable names makes them more difficult to read.

***We agree in principle, but we wanted to keep all the variable names as self-explanatory as possible, so we keep things intuitive for a general (non-mathematical) audience. We added an explanation about the methods IDs on page 13.***

6) 132: the important concept of calibration is used here, before being explained in detail in section 2.1.2

***The paragraph was reformulated to avoid using calibration, accuracy, and informativeness.***

7) 166: I suggest authors to provide some reference to a prior work on ROC curve definitions.

***The following reference was added to the paper***

***Fawcett, Tom (2006). "An Introduction to ROC Analysis"(PDF). Pattern Recognition Letters. 27 (8): 861–874.*** [***doi***](https://en.wikipedia.org/wiki/Doi_(identifier))***:***[***10.1016/j.patrec.2005.10.010***](https://doi.org/10.1016%2Fj.patrec.2005.10.010)***.***

8) 177: In this sentence, I think the word "Calibration" could be replaced by "Accuracy" and it will be valid. The explanation provided for calibration later in this paragraph is excellent, but I found that sentence a little bit confusing. Is "Calibration" the same thing as "Accuracy" ?

***Thank you! The text was clarified.***

9) 199: Where is the "Refinement" term ?

***We do not explicitly define the refinement term because we are not using it. We have clarified this in the text, on page 8.***

10) 202: Entropy is used here, very roughly defined, but it is defined in more detail later in Section 2.1.3.

***The text was modified and moved at the beginning of the next section.***

11) Section 2.1.3: The definition of Entropy in Equation (3) is a little bit confusing. I think it is a very important concept that should be defined more properly (in terms of having a more self-contained manuscript). I would suggest to cite and link with this article "A modified belief entropy in Dempster-Shafer framework, Zhou 2017" and provide the Shannon Entropy definition used in that article (which you could later modify to this particular problem of having the entropy of the particular distribution (pk, 1-pk).

***The definition of Entropy from what was eq 3 in the previous version of the manuscript was Shannon’s Entropy for a binary variable. The definition presented in "A modified belief entropy in Dempster-Shafer framework, Zhou 2017" is the same, but for variables with more than 2 possible states. However, since entropy is not used in the analysis we have removed the equation.***

12) Section 3: The idea of seed questions could be linked to Transfer Learning (as mentioned earlier) as well as with Supervised Learning.

***A link to transfer learning was added in the Introduction (see answer to comment 4).***

13) 297: I suggest Authors to add Bi,c to "simply takes the average of the best estimates B i,c of each individual)" for clarity.

***Thank you! We have added B_i,c_ to the text.***

14) 301: Why in this line "the mean" (i.e. I assume arithmetic mean) is different from the "The simple Average.." in line 299.

***The text was reformulated for clarity.***

15) 318: The concept of "extremizes the mean estimate" I believe that it could be further defined and clarified (it is used again in line 692).

***Clarifications and a figure to better explain the concept of extremizing were added on pages 16 and 17 (figure 1) .***

16) 358: I think the name "KitchSinkWAgg" is somehow unexpected in this context, and I think it could be further clarified (I was totally curious about that). Additionally and more broadly, I think the manuscript is going to be improved if an extra table with all the different methods are summarized and described (what aspect of the proxies are each one of them trying to emphasize).

***"KitchSinkWAgg is a playful name that describes a mixture of methods. We have emphasized this same in the text on page 20.***

***Thank you for the suggestion. We have added Table 1.***

17) 438: I think the NVivo software could be referenced (if there is any reference), or at least clarified something else about it.

***A link and a reference to NVivo were added in the Supporting information, where the details of the methods have been relocated.***

18) 527: There is a broken reference in the manuscript.

***Fixed.***

19) 658: I am not sure what exactly the pronoun "Its" is referring to in this sentence.

***The text was changed to improve clarity.***

20) Figure 2: I think you have 22 methods, not 21.

***The numbering was wrong, and it is now fixed. Thank you for pointing this out.***

21) One last word regarding the Discussion section, it was hard for me to state clearly what are the findings of this work. Perhaps, stating the findings, one by one, in a clear manner helps readers to identify them.

***The Evaluations and Comparisons and the Discussion sections have been modified and extended.***

**Reviewer #2**

This study reports a pure experimental analysis on existing methods of information aggregation. 22 aggregation methods are examined with several data sets. The verifications rely on three criteria including accuracy, calibration, and informativeness. The evaluation results are clear to me: the beta-transformed arithmetic mean is the best, and Arithmetic mean of the non-parametric distribution is the worst. The statement is clear and complete. I enjoyed reading this paper. The present results are convincing to me.

***Thank you!***

**Reviewer #3**

The authors consider the problem of aggregating experts’ probability predictions of a future binary outcome. They consider many different aggregators that derive from the weighted average and differ in the way the weights are calculated. They apply the aggregators to three real-world datasets and compare the performances of the aggregators in terms of several well-established criteria. Even though no single aggregator emerges as a clear winner, the beta-transformed arithmetic mean (BetaArMean) performs very well.

Overall, I enjoyed reading the paper, and I believe the literature would benefit from an extensive comparison of different weighting schemes. However, the paper needs work before being published. I have listed below both general and specific comments. I hope they help the authors to improve this paper.

***Thank you!***

# General Comments

1. Many of the weighting schemes are stated without providing any rationale or evidence from pre- vious literature that such a scheme could be reasonable. The authors should put in more effort to explain the intuition behind each scheme and, whenever possible, cite past work that has argued for such a scheme.

***In our revision, we have made a concerted effort to present the proposed aggregation methods in the context of the existing literature. When no references for our hypotheses were available we’ve discussed the intuition behind the methods.***

2. The framing needs work. Currently the text is 33 pages long and feels like a long enumeration of different weighting schemes. Furthermore, the discussion and results only take up around 5 pages. Perhaps the following could help:
   (a) Introduction: Include a paragraph or two clearly stating what the goal of this study is. One idea is to state that unequal weights based on seed variables have been found to improve averaging in the context of point forecasts. This article now studies whether the result holds under probability forecasts. This is roughly what you already state in the beginning of Section 3. This statement, however, should come much earlier in the paper.

***The Introduction section was modified and extended to address this comment. The text on pages 4 and 5 clarify the context and goal of the study.***

(b) Discussion: What is the main take-away or discovery in this paper? If you decide to use the study goal proposed above, then you should link the results and discussion to that. For instance, can you say anything about why different weighting schemes make little difference in the context of probability predictions? What makes this inherently different from point predictions? Can you propose a solution for making the probability forecasting context more like the point forecasting context?

***The Evaluations and Comparisons, and the Discussion sections have been modified and extended. We hope they now address many of the issues above.***

# Specific Comments

3. Line 75: The authors could consider the work on *the Wisdom of the Inner Crowd* that has found improved accuracy from averaging multiple predictions from the same individual. See, e.g., Herzog and Hertwig (2014).

***Thank you! The discussion on page 3 (lines 77 to 85) includes the suggested reference.***

4. Line 151: There is a typo: ”outcomr”

***Fixed.***

5. Line 151: The authors could give context by stating that a score of 0 implies perfect accuracy and

0.5 is guaranteed by constantly predicting 0.5 for all future events.

***Text was added to clarify this.***

6. Lines 174-175: The authors mention ”arbitrary probability threshold.” Can you explain what this means? The AUC requires a threshold (e.g., 0.5) above which the event is taken to occur and below which it is taken to not occur. How is this done exactly in your case?

***The text was wrong; AUC does not require a threshold. We have corrected and clarified the explanation on page 7.***

7. Equation (4): Can you define *I*(*pk,* 0*.*5)? Perhaps I missed it.

***Thank you for pointing out the missing formula. We have now added it on page 9.***

8. Lines 251-255: The authors claim that more seed variables are needed when probabilities are elicited. Can you provide intuition for this? My intuition says the opposite: by asking the experts’ for their full distribution – not just a point estimate – I gain more information about their true ability per prediction. This suggests that I would need fewer – not more – seed variables.

***This is a misunderstanding because of the poor explanations from the previous version of the manuscript. The text on page 4 was modified and clarified.***

9. Lines 269: The phrase ”In formulae where we single out claim *c*, we will have claims indexed by *d*, for *d* = 1*, . . . , C*” is confusing. First you index claims by *c*, but then also mention *d*. Perhaps a simple (one sentence) example can help here to clarify.

***The text was modified and moved (together with all technical details) to Section 6.***

10. Lines 275: The authors should consider adding a table with summary statistics of the data. For instance, this could include the number of claims, number of experts, number of predictions per claim, the number of claims that were true (a.k.a., the base rate).

***Thank you for this suggestion. Table 2 was added on page 26.***

11. Section 2.9: The weighted average of probability prediction is known to lack both calibration and sharpness even if the individual probabilities are calibrated. Ranjan and Gneiting (2010) show this for fixed weights. Satopaa (2017) generalize the result to random weights. This sub-optimality then led to the development of the extremizing algorithms (see, e.g., Satopaa et al. 2014; Baron et al. 2014). The authors should, in particular, review Baron et al. (2014) because it can help them motivate the median and some of the other aggregators. Now, these sub-optimality results are theoretical and they do not imply that the weighted average can never be useful. There are papers arguing the equally weighted average is “robust” in the sense that it does not rely on unstable parameter estimation (e.g., Jose and Winkler 2008). As a result, it can be useful. Finally, it is interesting that the authors do not cite Ranjan and Gneiting (2010) even though, I believe, this is where the BetaArMean aggregator was originally proposed.

***Thank you for the wealth of suggestions in terms of discussion points and references. The paper was reorganized to discuss all of the above and to mention most of the proposed references in Section 3.2.***

12. Line 310: Sometimes the probit instead of the logit is used. These two link functions are, however, similar when the predictions are close to 0.5. But both become infinitely large or small if the probabilities are 1.0 or 0.0. How did the authors handle such extreme predictions?

***Clarifications were added at the bottom of page 15 and the top of page 16.***

13. Line 313: To what papers are the authors referring here? Can you include citations?

***Reference added.***

14. Line 337: The authors could provide more intuition or citations to back up some of the weight- ing schemes. For instance, what is the intuition behind IndIntWAgg? It seems that it could be argued on the grounds of individuals having different perceptions of the probability scale. Is this accurate? Second, consider AsymWagg. Why is asymmetry a sign of certainty? Any citations supporting this claim?

***We are not aware of any specific literature that might underpin IndIntWAgg and AsymWAgg. These are based on our own intuition, i.e., for IndIntWAgg, we intuit that people who vary their interval width over different questions are thinking more carefully about their bounds and reflecting on their confidence on that particular question, relative to questions they’d answered previously. For AsymWAgg, we intuit that that participants who place their best estimate away from the middle of their uncertainty interval may have more thoughtfully considered the balance of evidence they entertained when producing their upper and lower bounds. We have added these justifications to the revised manuscript (pages 19,20).***

15. Lines 368-370: This sentence is confusing. I believe *r* = 0*.*33 is the correlation coefficient but based on the given wording I cannot be sure.

***We have reformulated the text to enhance clarity.***

16. Line 402: Why limit to granularity threshold of 0.05? The authors could consider several levels of granularity: 0.1, 0.05, and 0.01. The more granular the prediction, the more weight it gets.

***A 0.1 granularity is a given (there cannot be a coarser scale). Very few participants (less than 30 percent for 2 out of the 3 data sets) used finer scales. As a consequence, for a granularity of 0.01 most weights will be zero (79% for ACE and 72% for SIPS), so most participants will be left out of the analysis.***

17. Line 427: Does the number of reasons correlate with accuracy?

***Thank you for this comment. The number of reasons does correlate with accuracy. We now mention this on page 22.***

18. Lines 423-434: What is ICR? Can the authors explain this part more carefully?

***The text is now changed and relocated to the Supporting information section.***

19. Line 470: Does this mean that the accuracy scores (Brier, AUC, etc.) shown for QuizWAgg are based on different set of outcomes than the rest of the aggregators?

***No, it simply means that the weights are calculated using the quiz scores. Nothing changes for the calculation of the accuracy scores.***

20. Line 481: What is the rationale for giving the points in this manner?

***The quiz tested participants on their knowledge and understanding of statistical concepts (12 questions) and meta-research (10 questions). Questions that required less effort to answer (i.e., 10 true/false questions and one two-part question) were assigned half points. We have added this information to the revised manuscript on page 52.***

21. Line 504: The authors could use a figure to illustrate *F_i_*(*x*).

***Added figure 2 on page 18.***

22. Line 509: Can you explain why you have chosen this form for *σ_i,c_*? What is the motivation for it?

***An explanation of the variance was added on page 54.***

23. Line 520: Instead of calculating the posterior mean of *µc* and then transforming it to the unit interval, you could propagate the posterior uncertainty to the aggregate *p*ˆ*c*. In other words, the proper Bayesian way of doing this would compute *p*ˆ*c* for each one of the posterior draws in your MCMC sample and then use the mean of those values as the final aggregate.

***We believe that performing the transformation before or after the sampling will not significantly affect the resulting Bayesian updating.***

24. Line 527: The ”[link to repository]” does not seem work.

***A reference to a preprint was added.***

25. Move Section 4 before Section 2. This way you can use the data as a motivation, and the descriptions of the aggregators would make more sense as well.

***Done.***

26. Have the authors made the repliCATS and ACE-IDEA datasets publicly available? I did not see a mention of this in the paper.

***The datasets are available on OSF (https://osf.io/hkmv3/). We now mention this before introducing the data (Section 3, page 10).***

27. Line 622: The GJP also considered teams (both trained and untrained) and superteams. Each team had around 5 individuals. In the publicly available GJP dataset, the untrained teams are indicated by condition 4a; the untrained teams with condition 4b; and the superteams with condi- tion 5. Perhaps it makes more sense to analyze these groups rather than “random groups” of 10 individuals.

***We have added text on page 13 to explain the reasons behind the random subsets of GJP data. Mainly we wanted to have a dataset with properties as similar to the IDEA-ACE dataset as possible.***

28. In the ACE tournament the experts were allowed to make and update their predictions until the events resolved. Of course, predicting an event 3 months from now is much harder than something that will happen tomorrow. How did the authors take into account the different time horizons of the predictions? As a suggestion, they may consider using predictions made 30 days before the event resolutions, as was done in Satopaa et al. (2021).

***We did not account for the different time frames, mostly because in the replication project a time frame is harder to define and use in such analysis. We have briefly mentioned this when we discuss the differences between datasets analyzed in this paper (on page 26).***

29. Figure 1: Could the authors comment on the statistical significance of these differences? For instance, the repliCATS is a very small dataset. Therefore it is likely that none of these differences are statistically significant.

***None of these differences are statistically significant; we have now clarified this in the text.***

30. There are some inconsistencies in the discussion. For instance, on line 673 the authors state that “there are very few reasons to proclaim one aggregation method better than another.” But then on line 690 the BetaArMean is declared as a “clear winner.”

***The Discussion section has changed considerably, and it is now consistent with the Evaluations and comparisons section.***

31. Lines 712-720: I liked this discussion on the median. There are several papers arguing that the median performs well. For instance, consider Hora et al. (2013) and Han and Budescu (2019). Perhaps these references can help the authors to strengthen this result.

***Thank you for this suggestion! We have incorporated this point (and the suggested references) in the Discussion section, on page 34 .***

32. An interesting future study could compare the different types of *augmented elicitation* schemes where the decision-maker asks the experts to provide more than just the prediction of the outcome. Recent references are Prelec et al. (2017); Palley and Soll (2019); Palley and Satopaa (2020); Martinie et al. (2020).

***The Discussion section includes a paragraph on the above (page 33).***

**Reviewer #4**

I was hoping to gain some new insights into aggregation when I read this paper. I wish I had. This paper seems more like a report to a government agency than a journal article that tells a story about how to improve aggregation.

1) Many of the methods considered seem way too similar to matter, and yet they are considered anyway.

***The description, details, and motivation for the chosen aggregation methods were extended and improved. If “too similar” refers to the methods’ formulation, we hope we have now clarified the differences. If “too similar” refers to their performance, we agree, but there is little we can do about that. The methods were proposed prior to evaluating them (in the hope that they won’t have a similar performance) and we simply report on the performance of all proposed methods.***

2) The writing is difficult to follow.

***The text was reorganized and extended to improve clarity.***

3) and the graphs are too small to show the differences the authors claim they find – the best and worst aggregation rules across datasets. I don’t see a good explanation for WHY these aggregation rules are the best and worst.

***The figures were modified for better visibility; an extra figure with numerical values was added, and more clarifications were added in the text of the Evaluations and Comparisons section***.

4) I don’t understand what it was about the data sets that may had produced the differences, and I’m just not learning enough from the paper.

***Extra text was added on pages 24 and 26; Table 2, summarizing some of the most important differences between the datasets, was also added on page 26.***

5) Perhaps I could be convinced that a very different version of this paper could be published. The new version would have to get right to the point and focus on the key points – not a laundry list of aggregation rules.

***The Introduction and the section presenting the aggregation methods have changed considerably to improve readability and clarity. We have added a summary table of the methods and moved all the mathematical details in the Supporting information section.***

6) It would also have to include an explanation of why the results came out the way they did. Presentation of the arguments is not strong in the current form.

***The Evaluations and Comparisons and the Discussion sections have been modified and extended.***

7) Figures 1 and 2 compare too many rules, and they don’t really highlight the main points of the story.

***A third figure was added, and the text was extended for clarity.***

8) Sorry to be negative, but I think the authors should focus more on what they add to the literature that is different, new and will result in progress on these issues.

***In our revision, we have made a concerted effort to present the results in the context of the literature and believe that the novelty and relevance of this research is now clearer.***
