# Supplementary material for: Mathematically aggregating experts’ predictions of possible futures
Source: PLoS One. 2021 Sep 2;16(9):e0256919. doi: 10.1371/journal.pone.0256919 (PMC8412308; doi:10.1371/journal.pone.0256919)
Supplement: Attachment — Submitted filename: Review_final.docx [file pone.0256919.s005.docx]

***Reviewer #1***: All the issues that I previously pointed out were effectively addressed. Indeed, the manuscript changed quite a lot and now the exposition of the ideas is much more clear.

The included sections really help to understand the basis of the different methods, and there are now several references that truly aid in supporting the statements.

Additionally, now the section 3.2.1 really explains in a better way, step by step, all the different methods and the rationale behind them. The table 1 offers a clear overview and categorization of all of them. Now the take-home message (e.g. extremizing he aggregate through the BetaArMean) is much more clear.

The idea of moving the burden of the details of the different methods to Section 6, improves a lot the structure of the manuscript: it is now easier to read.

*Some final suggestions: Figure 1, the scale font is completely unreadable (compare the fonts to the ones that you have on Figure 2 which can be perfectly read).*

The manuscript is now lengthy, but I believe it fits better what is trying to do: summarizing different methods of mathematically aggregation of decisions from human experts.

***Thank you! We have modified Figure 1 so that the axis tick values are now readable.***

***Reviewer #2:***  This is my second-round reviews on this paper. Again, I enjoyed reading this paper. Compared with its previous version, some parts have been further clarified.

This paper provides a comprehensive review of methods on information aggregation, in a scenario of group (experts) decision-making. Although the studies are purely empirical, which may lead to the lack of theoretical background and profundity, the concrete contribution on applications has been impressive and applaudable. Therefore, again, I would like to recommend acceptance of this paper.

*For a minor issue, I would suggest the authors revise the abstract. The current abstract contains too much background (motivation, research gap, etc.), and yet, only three sentences (lines 20-24 page 1) regarding your substantial works of this paper. It is better to mention your works, your contribution, and the significance of this study in the abstract.*

***Thank you! We have modified the abstract to better capture the contribution of this paper to the field.***

***Reviewer #3:*** (Comments submitted as a separate file)

1. I like how the authors have brainstormed for many different weighting schemes and how they then compare the aggregators on several datasets on important applications. However, having read the paper a few times now, I am still left thinking:” What have I learned? What do I know now that I did not know before?” I would encourage the authors to add in the discussion section one paragraph that states this clearly.

***We have modified the abstract and added a new paragraph to the Discussion section to better summarise the paper’s key messages.***

2. Figure 2 and Table 2 are great new additions!

***Thank you!***

3. The final paragraph on page 4. The authors explain how probability predictions require more seed variables than quantile predictions. I had an earlier comment on this as well, and unfortunately it is still not entirely clear to me. Is it because with probability predictions a forecaster reports only one quantity per outcome, whereas with quantile predictions the forecaster reports multiple quantities (the quantiles at given cutoffs), leading to more stable evaluation of accuracy? I hope the authors can help me understand this better, either in response or in the paper.

***When addressing this comment in the previous round of reviews we only added*** *“This is a consequence of the instability of calibration and accuracy measures proposed for evaluating elicited probabilities (Hanea and Nane 2019).”* ***Although we agree this is not clear enough, we avoided lengthening the (already quite long) paper.***

***Hanea & Nane discuss and explore this issue in their paper from 2019,*** *Calibrating experts' probabilistic assessments for improved probabilistic predictions.*

***Earlier papers looking at the instabilities of performance scores when eliciting discrete distributions are (referenced within the above-mentioned paper)***

***Cooke, R.M., 1991. Experts in Uncertainty: Opinion and Subjective Probability in Science.***

***Environmental Ethics and Science Policy Series. Oxford University Press.***

***Cooke, R.M., Mendel, M., Thijs, W., 1988. Calibration and information in expert resolution.***

***Automatica 24 (1), 87–94.***

4. Page 8, line 215: I thought this was the definition of calibration/reliability – not of resolution.

***Thank you for pointing this out. We have now changed the text to correctly explain the resolution term.***

5. Page 13, lines 334-335: It seems that the authors use each forecaster’s final prediction of an out- come. In the GJP dataset, some forecasters updated frequently, some very little. Some questions were open for months, others for weeks. The authors way of choosing the predictions can result in situations where one forecaster’s prediction was made months before the event resolution (with high amounts of uncertainty) while another forecaster’s prediction was made a day before the event resolution (when almost no uncertainty remains). Clearly the forecasts were made under very different circumstances. I made an earlier comment on this as well and suggested that the authors would use a 30-day horizon. The authors, however, chose not do this because in the replication study time is difficult to define and use. Unfortunately, I am not convinced by this argument. The different studies use different questions/contexts anyways. I assume that in the replication study the forecasters made predictions under roughly similar levels of uncertainty. Shouldn’t the authors seek to control for the level of uncertainty in their analysis of the GJP data as well?

***We appreciate the reviewer’s concern about the different timeframes and the need to control for as many elements as possible. However, eliciting the probability of research replication has no clear timeframe, and the only way we can incorporate the uncertainty in predictions by eliciting bounds. In the previous review we have considered this issue seriously and for a variety of reasons decided to not control for the different timeframes. The main reason is is the one mentioned above. Another reason is that previous analysis made by the authors of (economic) predictions showed little variation when controlled for a 3 months vs 6 months’ time-frame. Another reason is the similarity between the ACE data and the GJP data: in that both cases we choose to take the final prediction, even though the time of the prediction may differ. We assumed the final prediction is the one made after the most thinking and evaluating of available information. For the datasets elicited with the IDEA protocol we have a measure of uncertainty associated with the predictions provided by the bounds. GJP dataset doesn’t contain this sort of info. The last reason was the difficulty of working with the GJP data set, due to the lack of clear descriptions of the dataset fields.***

6. I like how the authors now group the aggregators. This is very helpful. The authors could also discuss how the aggregators have different data requirements. For instance, extremization needs past performance data, whereas the median does not. Therefore, it is not surprising to see that extremization performs better than the median. Conditional on similar data requirements and hence breadth of applications, which aggregators seem to perform the best? This could be added, e.g., into the discussion.

***We have added a few words about this in the Discussion section.***
